# Cross-cultural adaptation of the Thoracic Outlet Syndrome Index for Turkish-speaking patients with thoracic outlet syndrome

**DOI:** 10.55730/1300-0144.5824

**Published:** 2024-04-03

**Authors:** Hakkı Çağdaş BASAT, Fatih ÖZYURT, Muhammed İhsan KODAK, Gülşah ÖZSOY, Caner KARARTI

**Affiliations:** 1 Department of Orthopedics and Traumatology, Faculty of Medicine, Kırşehir Ahi Evran University, Kırşehir, Turkiye; 2 Department of Physiotherapy and Rehabilitation, School of Physical Therapy and Rehabilitation, Kırşehir Ahi Evran University, Kırşehir, Turkiye; 3 Department of Physiotherapy and Rehabilitation, Faculty of Health Sciences, Selçuk University, Konya, Turkiye

**Keywords:** Thoracic outlet syndrome index, scale, cross-cultural adaptation

## Abstract

**Background/aim:**

Considering that there is no specifically designed scale to measure quality of life (QoL) and level of functioning among Turkish-speaking patients with thoracic outlet syndrome (TOS), the aim of this study was to adapt the Thoracic Outlet Syndrome Index (TOSI) to the Turkish language (TOSI-TR) and analyze its psychometric properties in patients with TOS.

**Materials and methods:**

Thirty-nine patients with symptomatic TOS were included in the study. The participants were assessed using the following measures: the QuickDASH, the Western Ontario Rotator Cuff Index (WORC), and a visual analog scale (VAS). The psychometric properties of the TOSI-TR were examined in terms of test–retest reliability, construct validity, convergent validity, and feasibility.

**Results:**

The ICC_2,1_ of the TOSI-TR was 0.949 (95% CI: 0.903–0.973). The Kaiser–Meyer–Olkin value was found to be 0.716 with a significant result for Bartlett’s sphericity test (p < 0.001). The TOSI-TR had a one-factor solution explaining 74.05% of the total variance. There was a significant correlation between TOSI-TR scores and QuickDASH (r = 0.762, p < 0.001), WORC (r =0.794, p < 0.001), and VAS (r = 0.663, p < 0.001) scores. The WORC and VAS scores were significant determinants of the TOSI-TR score, explaining 65.3% of the variance. There were no floor or ceiling effects.

**Conclusion:**

The TOSI-TR is a reliable, valid, and feasible questionnaire for the QoL and functional status in Turkish-speaking patients with TOS. We recommend this 15-item scale for optimal assessment in patients with TOS.

## 1. Introduction

Thoracic outlet syndrome (TOS) refers to a group of symptoms caused by compression of the neural and vascular anatomical structures in the thoracic outlet [1]. The symptoms include a sensation of numbness and tingling, chronic pain in the head and neck, and pain and weakness in the upper extremities [2]. Although TOS is a treatable condition, patients suffer from pain, dysfunction, anxiety, and depression due to the challenges of obtaining a correct diagnosis [3]. If not treated successfully, the syndrome can cause significant functional, psychological, and social impairment, leading to loss of employment and deterioration in quality of life (QoL) [1,2].

The measurement of QoL is particularly helpful in syndromes with complexity of presentation, such as TOS [4]. Although there is no specific QoL instrument for TOS, upper extremity and neck measures allow its indirect measurement. The Disabilities of the Arm, Shoulder, and Hand (DASH) Questionnaire [5], the Western Ontario Rotator Cuff Index (WORC) [6], and the Cervical Brachial Symptom Questionnaire (CBSQ) [7] are tools that have been specifically designed to measure the symptoms and function of patients with disorders of the upper extremities and the cervical spine.

Based on previous self-report tools used in patients with TOS, Vastamäki et al. designed a new questionnaire for patients with TOS known as the Thoracic Outlet Syndrome Index (TOSI) [4]. The authors proposed a 15-item TOSI questionnaire, rated on a scale of 0–10, to assess QoL and function in patients with TOS by measuring pain and physical symptoms (7 items), sports and recreation (2 items), work (4 items), lifestyle (1 item), and emotions (1 item). The questionnaire included items of the WORC, the QuickDASH, and the CBSQ, all of which showed acceptable face, content, and construct validity and internal consistency [4].

A more in-depth clinical and radiological evaluation is required to determine the optimal therapeutic options for enhancing the QoL and functioning of patients with TOS [8]. Considering that there is no specifically designed scale to measure the QoL and level of functioning of Turkish-speaking patients with TOS, the aim of this study was to adapt the TOSI to the Turkish language (TOSI-TR) and analyze its psychometric properties in patients with TOS.

## 2. Materials and methods

### 2.1. Translation process

Forward and backward translations were carried out in accordance with international recommendations [9]. Initially, a physiotherapist and a specialist in English translation translated the TOSI questionnaire from English into Turkish. To discuss the Turkish version, a meeting was organized between the translators and the research team. In addition, two native English speakers fluent in Turkish conducted the back-translation. They were not familiar with the original TOSI. A panel of experts, including all the members of the team involved, then designed the prefinal Turkish version (TOSI-TR), which was tested on a sample of 10 patients with TOS to assess its ease of reading, clarity, and required time. Those sample patients continued to be included in the study and their data were analyzed. The tools, subjects, and methods used in the study phases are detailed below.

### 2.2. Participants

Thirty-nine patients with symptomatic TOS (sTOS) who were admitted to the Orthopedic and Traumatology Clinic were included in this study. The diagnosis of sTOS was based on magnetic resonance imaging (MRI) and clinical assessment by an orthopedic surgeon (HÇB). All patients were then referred to the physiotherapy clinic for assessment and evaluated by an experienced physiotherapist (CK) for baseline and retest measures for TOS patients. The local ethics committee approved the study protocol (2023/463). Written and verbal informed consent was obtained from all participants.

Patients over the age of 18 who had experienced symptoms for at least 3 months and were diagnosed with sTOS were included in the study. The diagnosis was based on MRI findings, which included a restricted costoclavicular space, hypertrophy of the subclavius muscle, and a restricted retropectoralis minor space, and the presence of at least three out of four symptoms. These four symptoms included pain and tingling in the arm and hand; the worsening of symptoms when the arm was elevated; tenderness over the clavicle, pectoralis minor muscle, and brachial plexus; and a positive elevated arm stress test [10,11].

Patients were excluded if they had cervical radiculopathy or myelopathy, a history of cervical spine surgery, any inflammatory disease, upper extremity neuropathy, previous head or neck trauma, or a history of treatment in the previous 3 months [10].

### 2.3. Outcome measures

The TOSI is a questionnaire for disease-specific assessment of patients with TOS. It contains 15 items measuring 5 domains, each scored on a scale of 0–10 points where 0 is best and 10 is worst: pain and physical symptoms (7 items), sports and recreation (2 items), work (4 items), lifestyle (1 item), and emotions (1 item). The total score is calculated as the total of the 15 responses on the questionnaire, ranging from 0 to 150. The best possible score is 0, while the most severe TOS symptoms correspond to a score of 150 [4].

The participants were also assessed using the following reliable, valid, and culturally adapted measures: the QuickDASH questionnaire for the assessment of physical ability and symptoms of the upper extremities [4], and the WORC for the assessment of disease specific-quality of life [12].

### 2.4. Psychometric properties of the TOSI-TR

#### 2.4.1. Reliability study

For the test–retest assessment, the TOSI-TR questionnaire was administered again to all participants 7 days after the first assessment. It was ensured that the clinical characteristics of the patients were stable during this period [13].

#### 2.4.2. Validity study

Construct validity was examined using exploratory factor analysis (EFA) for the TOSI-TR questionnaire. The scree plot, the percentage of the variance explained by the factorial model, and the patterns of the factor loadings were examined [14]. To evaluate convergent validity, correlation coefficients were used to analyze the relationship between the TOSI-TR and the VAS, QuickDASH, and WORC.

#### 2.4.3. Feasibility

To check the feasibility of the measure, the amount of time spent by patients in completing the TOSI-TR questionnaire was noted. We also examined ceiling and floor effects, which are considered to be present when more than 15% of respondents receive the minimum or maximum score theoretically possible [15].

### 2.5. Sample size and statistics

G*Power Software (Version 3.1.9.2, Düsseldorf University, Düsseldorf, Germany) was used to calculate the sample size. Based on ICC hypothesis testing, with an alpha value of 0.01, statistical power of 0.90, lower limit ρ(0) of 0.7, and upper limit ρ(1) of 0.9, 39 subjects were required while taking into account an ICC_2,1_ value of 0.90 and a drop-out rate of 15% [16].

IBM SPSS Statistics 22.0 for Windows (IBM Corp., Armonk, NY, USA) was used for statistical analysis. Descriptive statistics were expressed as mean ± standard deviation (SD) for continuous variables and as ratios (%) for categorical variables [17]. The internal consistency and test–retest reliability of the TOSI-TR were assessed using Cronbach’s alpha and ICC_2,1_ with 95% confidence intervals (CIs), respectively [13]. To examine the visual agreement between the first and second TOSI-TR sessions, a Bland–Altman plot was created. The 95% CIs and 95% limits of agreement (LoA) are shown for the differences between the two measurements.

The standard error of measurement (SEM) and minimum detectable change (MDC) were also calculated. The formula for estimating SEM is SD × √(1 − R). Here, SD refers to the standard deviation obtained from the first assessment and R represents the reliability coefficient of the TOSI-TR questionnaire [13]. The MDC threshold value is calculated by multiplying the SEM by 1.96 × √2, where 1.96 corresponds to the 95% CI and √2 represents the measurement error associated with two measurements [13].

EFA was used to analyze the factor structure of the TOSI-TR. Principal component analysis with varimax rotation was carried out using the first TOSI-TR scores [18]. The sampling adequacy was deemed sufficient, with a Kaiser–Meyer–Olkin (KMO) value of 0.7–1.0, while Barlett’s test of sphericity showed significance at p < 0.001, indicating the usefulness of EFA for data analysis. The scree plot, an eigenvalue cutoff greater than 1.0, and at least 10% variance were also considered for factor extraction [18].

The associations between the TOSI-TR, QuickDASH, and WORC were examined using Pearson product-moment correlation coefficients. To identify the variables with the most significant effect on TOSI-TR scores in patients with TOS, stepwise multiple linear regression analysis was used. Before the regression analysis, variables significantly correlated with the TOSI-TR were included in the regression model, namely the QuickDASH, WORC, and VAS. Outliers were identified and treated using Cook’s distance and centered leverage values. The regression equation formula was obtained. The significance level was set at p < 0.05.

## 3. Results

### 3.1. Translation

There were no problems with the translation procedure. The final version of the TOSI-TR is provided in the Supplementary file.

### 3.2. Reliability

A total of 39 patients (mean age: 41.51 ± 11.24 years, 31 women, mean body mass index of 27.50 ± 4.42 kg/m^2^) with TOS were included in the study. Descriptive and clinical characteristics of the participants are presented in Table 1. The test–retest reliability of the TOSI-TR was found to be excellent (≥0.90). The ICC_2,1_ of the TOSI-TR was 0.949 (95% CI: 0.903–0.973). The SEM and MDC_95_ for the TOSI-TR were 5.20 and 14.413, respectively (Table 2). Bland–Altman analysis revealed that the difference between the test and retest measurements of the TOSI-TR had 95% LoA ranging from −20.172 to 20.428 (Figure).

### 3.3. Construct validity

The KMO value was found to be 0.716 with a significant result for Bartlett’s sphericity test [χ^2^(45) = 278.68] (p < 0.001) and the sample size was determined to be sufficient for EFA. It was concluded that the TOSI-TR, which consists of 15 items, has a one-factor solution explaining 74.05% of the total variance. Factor loadings after varimax rotation are shown in Table 3.

### 3.4. Convergent validity

There were significant correlations between TOSI-TR scores and QuickDASH (r = 0.762, p < 0.001), WORC (r = 0.794, p < 0.001), and VAS (r = 0.663, p < 0.001) scores. To identify the potential determinants of TOSI-TR scores, QuickDASH, WORC, and VAS scores were used as independent variables in a regression model. It was found that the WORC and VAS scores were significant and independent determinants of the TOSI-TR score, explaining 65.3% of the variance (Table 4).

The regression equation formula was obtained as follows: 19.520 + (0.387 × WORC score) + (3.216 × VAS score); adjusted R^2^ = 0.653.

### 3.5. Feasibility

The TOSI-TR questionnaire was completed by all participants in less than 5 min. None of the TOS patients met the maximum or minimum achievable scores, both overall and per item. Thus, there were no floor or ceiling effects.

## 4. Discussion

The aim of this study was to adapt the TOSI to the Turkish language and analyze its psychometric properties in patients with TOS. It was found that the TOSI-TR is a reliable, valid, and feasible questionnaire for Turkish-speaking patients with TOS.

There is currently no agreement on the scales used to assess the outcome of treatment or follow-up for patients with TOS. To date, the DASH questionnaire [19], WORC [4], and CBSQ [20] have been used for the assessment of the functional status of these patients. Although some items from these questionnaires were included in the development process of the original version of the TOSI [4], these questionnaires do not provide enough specific measurements related to the functional status of TOS patients. For example, while the TOSI assesses pain severity in the axilla, thorax, neck, or cheek, this dimension is not assessed in the scores of the previous measures. These scores have self-report and performance-based components and are applicable for use among patients with shoulder-brachial-cervical pathology (except for the WORC, which is rotator cuff-specific) regardless of their specific diagnosis. Therefore, they are not TOS-specific. Improving QoL and functional status is the main goal of designing a treatment strategy; therefore, disease-specific QoL questionnaires, such as the TOSI-TR, need to be considered as measurement outcomes for patients with TOS.

The original version of the TOSI was developed in 2020. However, to the best of our knowledge, there has not been any cross-cultural adaptation study of it in any language published to date. Therefore, it is difficult to compare our data with the relevant literature. Considering that some related studies could still be recruiting patients, since diagnosing TOS requires good clinical experience and detailed examination, it may take time for researchers to obtain sufficient sample sizes.

We found that the test–retest reliability of the TOSI-TR was excellent and there were significant correlations between the TOSI-TR and the QuickDASH, WORC, and VAS. In addition, it was found that the WORC and VAS were significant and independent determinants of TOSI-TR scores, explaining 65.3% of the variance. The DASH [19], QuickDASH [4], WORC [4], and VAS [19] have been used as standard measurements for TOS to date, although they are not disease-specific. There were discernable correlations of TOSI-TR scores with these measurements. The present results demonstrate that the internal consistency and convergent validity of the TOSI-TR are acceptable. Moreover, since the TOSI-TR is a self-reported disease-specific questionnaire, being a scale that is easily filled out is an important factor for the cooperation of the patient. We found that the time required to complete the TOSI-TR is less than 5 min. Furthermore, none of the TOS patients met the maximum or minimum achievable scores. These findings confirm that there is no floor or ceiling effect for the TOSI-TR and that it is a feasible measurement tool.

Finally, the KMO value was found to be higher than 0.70 and a one-factor solution was confirmed using EFA, which explained 74.05% of the total variance [18]. Considering that TOS is a rarely diagnosed pathology and in light of the KMO value, our sample size seems sufficient. Moreover, the factor solution obtained here is in line with that of the original version [4]. Thus, the current findings provide evidence of construct validity and validate the use of a summed score obtained by adding the points from each question to assess symptom severity. However, as there has been no other cross-cultural adaptation study of the TOSI, we were unable to compare our findings with the relevant literature.

There are several limitations to our study. First, the study did not consider the surgery schedules of the patients. The second limitation is the unequal numbers of male and female participants. Third, the education levels of the participants could be an important factor for the results. We did not record the participants’ educational status. To assess convergent validity, correlation coefficients were used to analyze the relationship between the TOSI-TR and the QuickDASH and WORC. The original version was developed using CSBQ in addition to these assessments. However, since a Turkish version of CSBQ has not been developed, we were unable to evaluate our patients using this questionnaire. This was a limitation. To minimize measurement-related differences, it is recommended that future cross-cultural adaptation studies of the TOSI questionnaire focus on these limitations.

In conclusion, the TOSI-TR is a reliable, valid, and feasible questionnaire for the assessment of QoL and functional status in Turkish-speaking patients with TOS. We recommend this 15-item scale for the optimal assessment of patients with TOS.

## Supplementary file

The final version of the Turkish translation of the TOSI-TR.



## Figures and Tables

**Figure:**
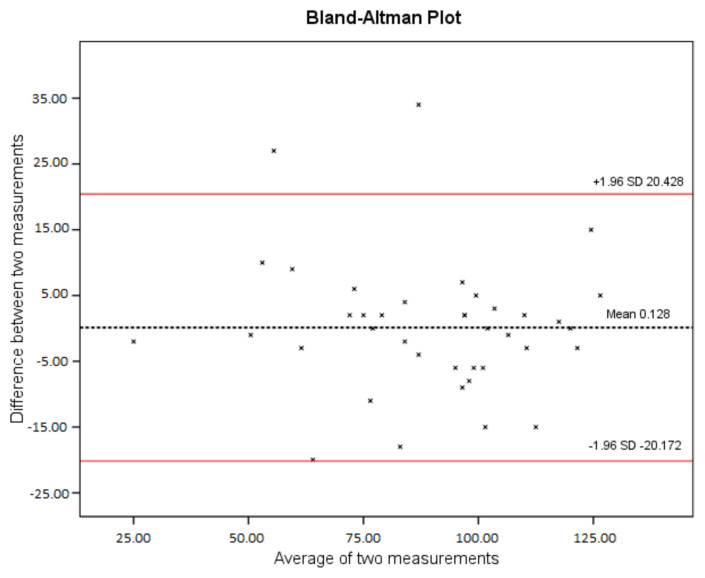
Bland–Altman plot.

**Table 1:** Descriptive and clinical characteristics of the participants.

Variable	Mean ± SD or n (%)
Age	41.51 ± 11.24
Body mass index (kg/m^2^)	27.50 ± 4.42
Female (%)	31 (79.5%)
Duration of pain (months)	17.41 ± 18.17
Pain during activity (VAS)	7.00 ± 1.87
TOSI-TR (first assessment)	89.36 ± 23.03
TOSI-TR (second assessment)	89.23 ± 23.97
QuickDASH	35.46 ± 7.30
WORC	122.33 ± 37.32

Abbreviations: VAS: Visual analog scale; TOSI-TR: Turkish version of the Thoracic Outlet Syndrome Index; DASH: Disabilities of the Arm, Shoulder, and Hand Questionnaire; WORC: Western Ontario Rotator Cuff Index.

**Table 2:** Test–retest reliability, standard error of measurement, and minimal detectable changes of the TOSI-TR.

	First assessment, mean ± SD	Second assessment, mean ± SD	ICC_2,1_ (95% CI)	SEM	MDC_95_
TOSI-TR	89.36 ± 23.03	89.23 ± 23.97	0.949 (0.903–0.973)	5.200	14.413

Abbreviations: TOSI-TR: Turkish version of the Thoracic Outlet Syndrome Index; ICC_2,1_: intraclass correlation coefficients with 95% confidence interval (CI); SEM: standard error of measurement; MDC: minimum detectable change.

**Table 3:** Factor loadings after varimax rotation.

TOSI-TR items	Item loading
1	0.749
2	0.601
3	0.587
4	0.614
5	0.691
6	0.511
7	0.505
8	0.697
9	0.663
10	0.711
11	0.692
12	0.714
13	0.763
14	0.593
15	0.653

TOSI-TR: Turkish version of the Thoracic Outlet Syndrome Index.

**Table 4:** Correlations between the TOSI-TR and QuickDASH, WORC, and VAS.

	QuickDASH	WORC	VAS
TOSI-TR	r = 0.762 p < 0.001^*^	r = 0.794 p < 0.001^*^	r = 0.663 p < 0.001^*^
Regression equation formula: 19.520 + (0.387 × WORC score) + (3.216 × VAS score); adjusted R^2^ = 0.653			

Abbreviations: TOSI-TR: Turkish version of the Thoracic Outlet Syndrome Index; DASH: Disabilities of the Arm, Shoulder, and Hand Questionnaire; WORC: Western Ontario Rotator Cuff Index; VAS: visual analog scale.

## Data Availability

The data that support the findings of this study are available from the corresponding author upon reasonable request.
