# Supplementary figures and images for: Cross-cultural adaptation of the Thoracic Outlet Syndrome Index for Turkish-speaking patients with thoracic outlet syndrome
Source: Turk J Med Sci. 2024 Apr 3;54(3):572–8. doi: 10.55730/1300-0144.5824 (PMC11265911; doi:10.55730/1300-0144.5824)

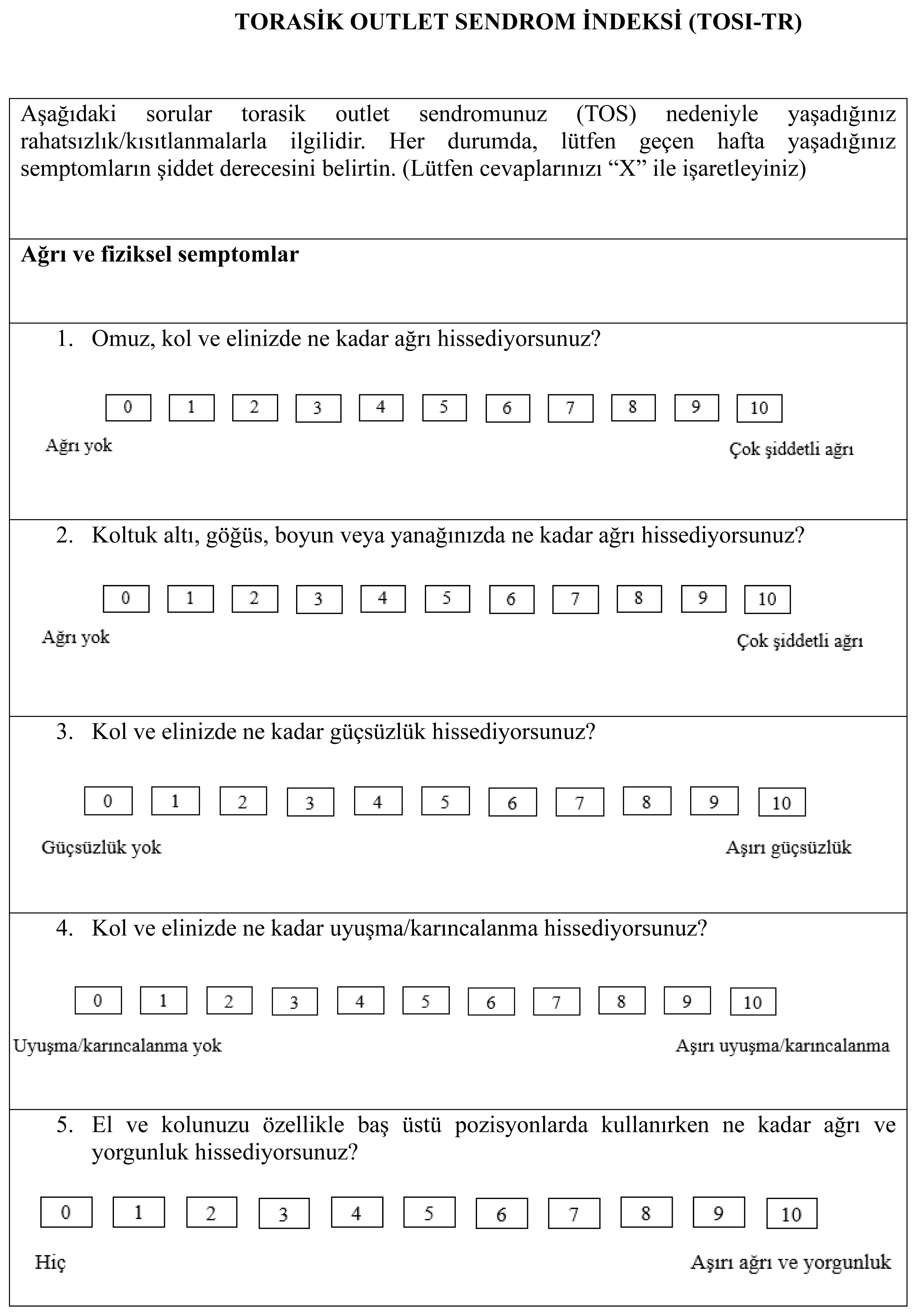

Supplement: Supplementary file 1 [file tjmed-54-03-572s1a.tif]

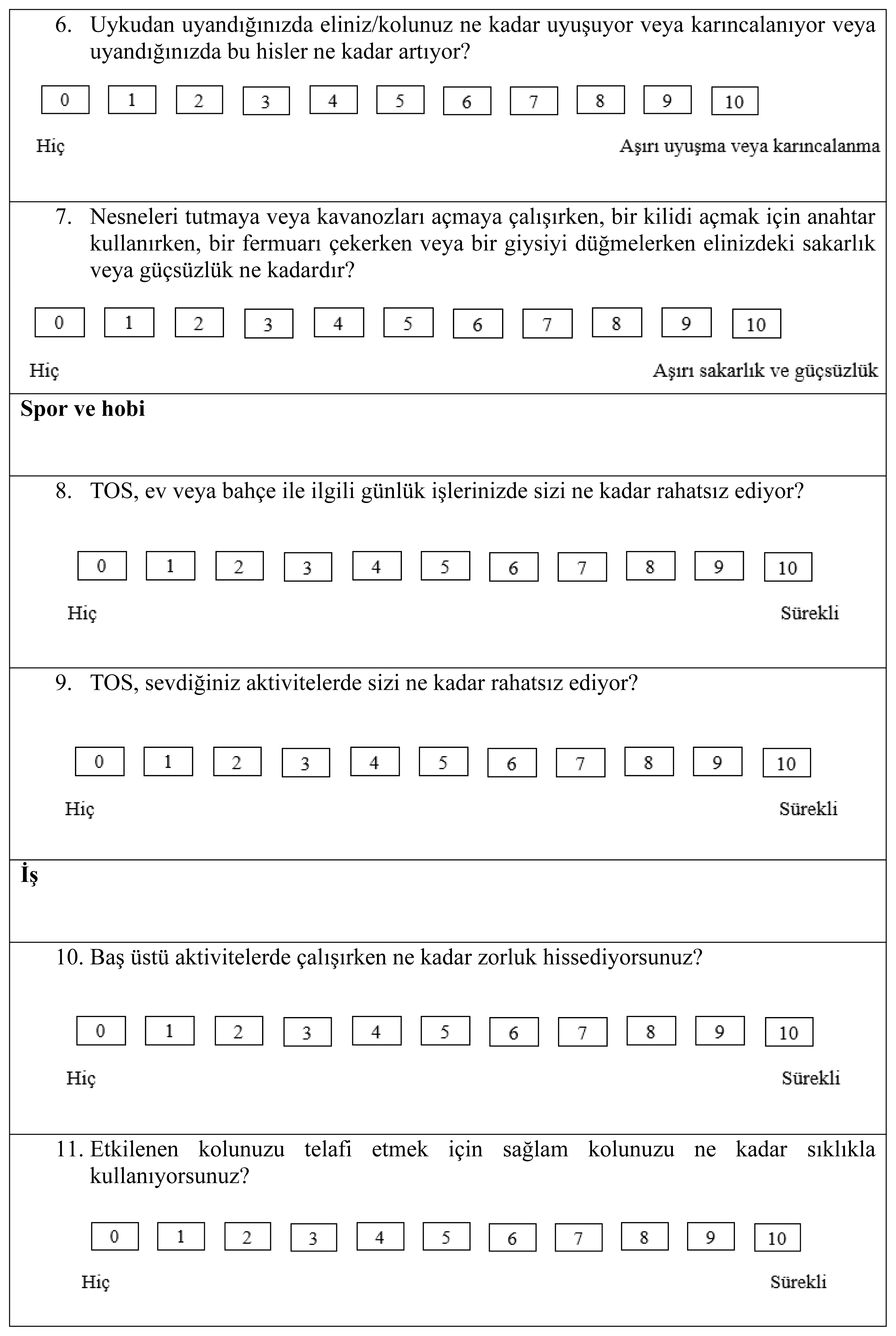

Supplement: Supplementary file 2 [file tjmed-54-03-572s1b.tif]

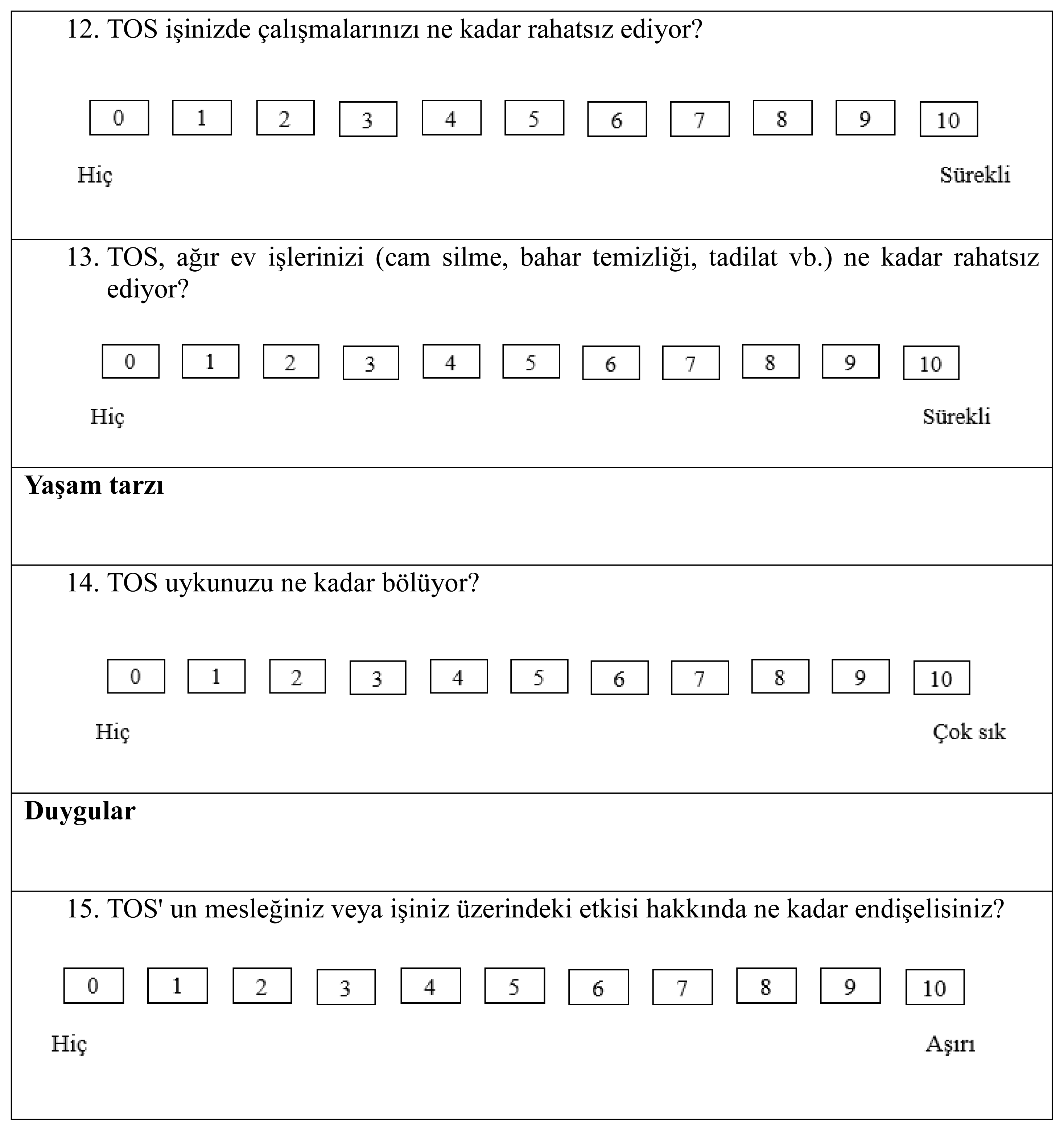

Supplement: Supplementary file 3 [file tjmed-54-03-572s1c.tif]
